# Three new species, *Xanthomonas hawaiiensis* sp. nov., *Stenotrophomonas aracearum* sp. nov., and *Stenotrophomonas oahuensis* sp. nov., isolated from the Araceae family

**DOI:** 10.3389/fmicb.2024.1356025

**Published:** 2024-04-09

**Authors:** Shu-Cheng Chuang, Shefali Dobhal, Anne M. Alvarez, Mohammad Arif

**Affiliations:** Department of Plant and Environmental Protection Sciences, University of Hawaiʻi at Mānoa, Honolulu, HI, United States

**Keywords:** Araceae, MLSA, pan-genome, *Xanthomonas hawaiiensis* sp. nov., *Stenotrophomonas aracearum* sp. nov., *Stenotrophomonas oahuensis* sp. nov.

## Abstract

*Xanthomonas* and *Stenotrophomonas* are closely related genera in the family Lysobacteraceae. In our previous study of aroid-associated bacterial strains, most strains isolated from anthurium and other aroids were reclassified as *X. phaseoli* and other *Xanthomonas* species. However, two strains isolated from *Spathiphyllum* and *Colocasia* were phylogenetically distant from other strains in the *Xanthomonas* clade and two strains isolated from *Anthurium* clustered within the *Stenotrophomonas* clade. Phylogenetic trees based on 16S rRNA and nine housekeeping genes placed the former strains with the type strain of *X. sacchari* from sugarcane and the latter strains with the type strain of *S. bentonitica* from bentonite. In pairwise comparisons with type strains, the overall genomic relatedness indices required delineation of new species; digital DNA–DNA hybridization and average nucleotide identity values were lower than 70 and 95%, respectively. Hence, three new species are proposed: *S. aracearum* sp. nov. and *S. oahuensis* sp. nov. for two strains from anthurium and *X. hawaiiensis* sp. nov. for the strains from spathiphyllum and colocasia, respectively. The genome size of *X. hawaiiensis* sp. nov. is ~4.88 Mbp and higher than *S. aracearum* sp. nov. (4.33 Mbp) and *S. oahuensis* sp. nov. (4.68 Mbp). Gene content analysis revealed 425 and 576 core genes present in 40 xanthomonads and 25 stenotrophomonads, respectively. The average number of unique genes in *Stenotrophomonas* spp. was higher than in *Xanthomonas* spp., implying higher genetic diversity in *Stenotrophomonas*.

## 1 Introduction

Genera *Xanthomonas* (Dowson, 1939) and *Stenotrophomonas* (Palleroni and Bradbury, 1993) are the groups of gram-negative and aerobic bacteria belonging to the family Lysobacteraceae (syn. Xanthomonadaceae) of Lysobacterales (syn. Xanthomonadales) order of Gammaproteobacteria class in the phylum Proteobacteria (Kumar et al., 2019; Parte et al., 2020; Bansal et al., 2021a,b; Bansal et al., 2023). *Stenotrophomonas* and *Xanthomonas* are phylogenetically closely related genera along with *Xylella* and *Pseudoxanthomonas* in Lysobacteraceae (Bansal et al., 2021b; Bansal et al., 2023). Before the current generic name, *Xanthomonas* was described as *Bacterium* in 1921 and later reclassified into the genus *Phytomonas* in 1923 (Doidge, 1921; Bergey et al., 1923; Dowson, 1939). The taxonomy of the first reported xanthomonad from pepper and tomato was changed several times, as described below. The pathogen was originally classified as *B. vesicatorium* and then *X. vesicatoria*; subsequently, it was given the trinomial pathovar *vesicatoria* first under *X. campestris* (Young et al., 1978) and later under *X. axonopodis* (Vauterin et al., 1995) and finally reclassified as a separate species, *X. euvesicatoria* (Jones et al., 2004; Constantin et al., 2016). Interestingly, before designating the genus *Stenotrophomonas*, the first stenotrophomonad isolated from pleural fluid of a hospitalized patient had been referred to as *X. maltophilia*; initially identified within the *Bacterium* genus, it was subsequently reclassified under *Pseudomonas* for a decade (Hugh and Ryschenkow, 1961; Swings et al., 1983; Palleroni and Bradbury, 1993). In 1993, due to distinct phylogenetic lineage from the other phytopathogens in *Xanthomonas*, *X. maltophilia* was replaced by *S. maltophilia* (Palleroni and Bradbury, 1993). At the time of writing this manuscript, there are 36 and 17 validly published species of *Xanthomonas* and *Stenotrophomonas*, respectively, as well as some other invalid species shown in quotation marks (““) throughout the rest of this article in the List of Prokaryotic names with Standing in Nomenclature (LPSN, last accessed on December 2022) (Parte et al., 2020).

Most *Xanthomonas* species are pathogenic to more than 400 different monocot and dicot plants, including economically important crops and ornamentals. Additionally, some *Xanthomonas* strains are non-pathogenic and associated with plants (Bradbury, 1984; Leyns et al., 1984; Vauterin et al., 2000; Ryan et al., 2011; Vandroemme et al., 2013; Parte et al., 2020; Timilsina et al., 2020; Mafakheri et al., 2022). On monocotyledonous hosts, *X. oryzae* pv. *oryzae* and *X. oryzae* pv. *oryzicola* are listed on the USDA Select Agent list, causing severe diseases of rice (*Oryza sativa*). In addition, *X. albilineans* causes leaf scorch of sugarcane (*Saccharum officinarum*), and *X. vasicola* is a causal agent of banana wilt (Ryan et al., 2011). Whereas *X. maliensis*, “*X. sontii*,” and “*X. indica*” were reported associated with the rice phytobiome (Triplett et al., 2015; Bansal et al., 2021a; Rana et al., 2022), *X. sacchari* was associated with sugarcane, causing rice sheath rot disease (Ivayani et al., 2023). Constantin et al. (2016) reclassified bacterial strains isolated from *Anthurium*, *Dieffenbachia*, and other ornamental Araceae plants into the species and/or pathovars, *X. phaseoli* including two pathovars *dieffenbachiae* and *syngonii*, *X. citri* pv. *aracearum* and *X. euvesicatoria*. The Araceae strains of *X. phaseoli* pv. *dieffenbachiae*, *X. phaseoli* pv. *syngonii*, and *X. citri* pv. *aracearum* were pathogenic on their original hosts, but *X. euvesicatoria* strains isolated from *Philodendron* caused weak symptoms and lacked host specificity on tested Aracea hosts (Constantin et al., 2017).

As for *Stenotrophomonas* spp., they are ubiquitous environmental bacteria isolated from various sources. The type species, *S. maltophilia*, is an opportunistic pathogen on humans infecting through clinical materials and equipment, and “*S. sepilia*” was isolated from a nosocomial patient’s blood specimen (Hugh and Ryschenkow, 1961; Al-Anazi and Al-Jasser, 2014; Gautam et al., 2021). In addition to the clinical species, *Stenotrophomonas* species are also from various sources, such as *S. humi* and *S. terrae* from soil, *S. daejeonensis* and *S. geniculata* from water, *S. pavanii*, *S. rhizophila*, and “*S. cyclobalanopsidis*” associated with plants, “*S. pennii*” and “*S. muris*” from animals, *S. lactitubi* and *S. indicatrix* from surfaces in contact with food, and *S. acidaminiphila* and *S. chelatiphaga* from sludges (Assih et al., 2002; Wolf, 2002; Heylen et al., 2007; Kaparullina et al., 2009; Lee et al., 2011; Ramos et al., 2011a; Weber et al., 2018; Bian et al., 2020; Gilroy et al., 2021; Afrizal et al., 2022). Notably, *S. maltophilia* was also encountered frequently in aquatic and plant-associated environments, and *S. rhizophila* strains isolated from the rhizosphere and geocaulosphere were separated from *S. maltophilia* based on 16S rDNA analysis and DNA–DNA hybridization data (Berg et al., 1996; Denton and Kerr, 1998; Minkwitz and Berg, 2001; Wolf, 2002).

Among monocot plants, the Araceae family includes the most economically important ornamental plants in Hawaii, especially the genus *Anthurium*. During the 1980s to 1990s, the anthurium industry was seriously damaged due to *X. phaseoli* pv. *dieffenbachiae* outbreaks (formerly called *X. axonopodis* pv. *dieffenbachiae*) (Alvarez et al., 2006; Constantin et al., 2016). Hundreds of bacterial strains were isolated from various plant genera in Araceae worldwide, including the strains collected during the outbreaks in Hawaii, and stored in the Pacific Bacterial Collection at the University of Hawaii at Manoa.^1^ In our previous five-gene multilocus sequence analysis (MLSA) of Lysobacteraceae strains isolated from the Araceae family, a strain from *Spathiphyllum* and another strain from *Colocasia* clustered within the *Xanthomonas* clade but formed a distinct monophyletic lineage, while two strains from *Anthurium* grouped with the *Stenotrophomonas* clade instead of the *Xanthomonas* clade (Chuang, 2023). Moreover, these two stenotrophomonads were distinct from the former two xanthomonads based on the utilizations of N-acetyl-D-galactosamine (GalNAc) and D-serine, and the inability to oxidize D-galactose, glycerol, pectin, and sucrose based on Biolog GEN III microplate assays (Chuang, 2023).

Hence, we sequenced the whole genomes of the former strains isolated from Araceae, which are potential novel species, comparing them with the genomes of *Xanthomonas* spp. and *Stenotrophomonas* spp. type strains. Based on the nine-gene MLSA, overall genomic relatedness index (OGRI) values, and pan-core genomic analyses, strains A6251^T^ from *Spathiphyllum* and A2111 from *Colocasia* are described as new species *X. hawaiiensis* sp. nov., strain A5588^T^ from *Anthurium* is described as *S. aracearum* sp. nov., and strain A5586^T^ from *Anthurium* is described as *S. oahuensis* sp. nov.

## 2 Materials and methods

### 2.1 Bacterial DNA isolation and genome sequencing

Bacteria were streaked out from the culture stock and grew on 2, 3, 5-triphenyltetrazolium chloride (TZC) agar medium (dextrose 5 gL^−1^, peptone 10 gL^−1^, 0.001% sterilized TZC, and agar 18 gL^−1^) at 28°C for 2 days. Bacterial genomic DNA was isolated from pure culture using QIAGEN Genomic-tip 100/G, following the manufacturer’s instruction (QIAGEN, Valencia, CA, USA). The Seqwell plexWell LP384 Library Preparation Kit and Native Barcoding Kit 24 V14 (SQK-NBD112.24) were used for barcode-indexed whole genome sequencing with Illumina NovaSeq system (Illumina San Diego, CA, USA) and Oxford Nanopore MinIoN Mk1C device (Oxford Nanopore Technologies, ONT, Oxford, UK), respectively. ONT long reads were base called and demultiplexed using basecaller and barcoder of GUPPY v6.3.2 on MANA, a high-performance computing cluster at the University of Hawaii at Manoa.

### 2.2 Hybrid genome assembly and genome annotation

The hybrid assembler, Unicycler v0.4.8 plugged in the web-server of BV-BRC 3.26.4,^2^ was employed by uploading paired-end (2 × 150 bp) Illumina short-read, high-accuracy basecalled ONT long reads for *de novo* genome assemblies (Wattam et al., 2017; Wick et al., 2017; Klair et al., 2022; Olson et al., 2023). In brief, Unicycler carried out SPAdes (v3.13.0) to assemble the Illumina short reads, and then, miniasm, minimap2 (v2.17), and Racon (v1.4.13) were run for long-read plus contig assembly, long-read bridging, and contig polishing, respectively. Alternatively, the genome of the strain (A2111) with a lower coverage of short reads was assembled by performing Flye v2.9.1 and genome assembly pipeline in the web server of BV-BRC 3.26.4 (see text footnote 2). Moreover, the genomic completeness and contamination were assessed by implementing the CheckM algorithm (Parks et al., 2015). The genome annotations were performed using Prokaryotic Genome Annotation Pipeline (PGAP v4.10) on NCBI (Tatusova et al., 2016) and Rapid Annotation using Subsystem Technology (RAST v2.0) web server (Aziz et al., 2008) as well.

### 2.3 Phylogenetic analyses

The partial 16S rRNA gene sequences of new species strains were amplified using primer set P16S-F1 (5’-AGACTCCTACGGGAGGCAGCA-3′) and P16S-R1 (5’-TTGACGTCATCCCC ACCTTCC-3′) by end-point PCR (Larrea-Sarmiento et al., 2019). Each 25 μL of PCR reaction mix contained 5 μL of 5X Q5 buffer, 5 μL of GC enhance, 2.5 μL of 5 μM primer F and R, 0.5 μL of 2.5 mM dNTPs, 0.5 μL of Q5 polymerase, 1 μL of gDNA, and 6.5 μL of nuclease-free water. The PCR reaction was run as follows: 10 s at 98°C; 35 cycles of 10 s denaturing at 98°C, 30 s of annealing at 58°C, and 30 s extending at 72°C; and 2 min at 72°C for final extension in a T100 Thermal Cycler (BIO-RAD Lab. Inc., Hercules, CA, USA). The sizes of PCR products were checked by running agarose gel electrophoresis, purified using Exo (exonuclease I)-SAP (shrimp alkaline phosphatase) method (GE Healthcare, Little Chalfont, UK) following the manufacturer’s instruction, and sent for Sanger sequencing service at the GENEWIZ company (South Plainfield, NJ, USA). The sequences were double checked with the assembled genomes.

For the phylogenetic analysis of 16S rRNA gene, the full-length sequences of 65 *Stenotrophomonas* and *Xanthomonas* species type strains were retrieved from their whole genome sequences and downloaded from GenBank on NCBI (Supplementary Table 1). The multiple alignment was performed using Geneious Prime 2021.2.2.^3^ The module of finding the best DNA/Protein model for the multiple alignment data was conducted, and the maximum likelihood (ML) phylogenetic tree was built using MEGA X (Kumar et al., 2018). The consistency of the phylogenetic tree was assessed by computing 1,000 bootstrapping analyses.

Additionally, the precise MLSA was performed to reveal the phylogenetic relations between the novel species and other *Stenotrophomonas* and *Xanthomonas* species. Nine housekeeping genes (*atpD*, *dnaA*, *dnaK*, *gltA*, *gyrB*, *nuoD*, *ppsA*, *rpoH*, and *uvrB*) used from the previous studies (Ramos et al., 2011b; Vasileuskaya-Schulz et al., 2011; Chuang, 2023) were retrieved from downloaded genomes. The sequences of the nine housekeeping genes were aligned with free end gaps algorithm separately. After trimming the both sequence ends of each gene, nine gene sequences were concatenated in alphabetic order using Geneious Prime for further analyzing. The ML phylogenetic tree was formed using MEGA X following the process as detailed above. The phylogenetic trees with bootstrapping analyses were created using web-based tool Interactive Tree Of Life (iTOL v6)^4^ (Letunic and Bork, 2021).

### 2.4 Genome similarity

To define new species, the pairwise comparisons of overall genomic relatedness indices (OGRIs) among the genomes of new species strains and other type strains of *Stenotrophomonas* and *Xanthomonas* species retrieved from NCBI database were calculated. The pairwise ANI and AP (alignment percentage) values were calculated using CLC Genomics Workbench 22.0.2 (CLC Bio-QIAGEN, Arahus, Denmark). Due to the inclusion of some incomplete genomes, OrthoANI (Average Nucleotide Identity by Orthology), which only considered the orthologous fragment pairs, was additionally calculated by performing Orthologous Average Nucleotide Identity tool (OAT) (Lee et al., 2016). Moreover, the pairwise dDDH values and the differences in G + C content (mol%) were inferred by estimating precise distance from whole genome sequences using the Genome-Genome Distance Calculator (GGDC) v3.0 on Type Strain Genome Server (TYGS) web server^5^ (Meier-Kolthoff et al., 2013, 2022).

### 2.5 Pan-genome analysis

Whole genome sequences of the new species and closely phylogenetically related species in each genus were used for pan-genome and core-genome analyses. Prokka v1.14.6 (Seemann, 2014) was used to re-annotate representative genomes, and the output gff files were used as input files for the Roary v3.13.0 pipeline (Page et al., 2015). For Roary, core and accessory genes were assessed with 80% minimum BLASTp identity, and multi-FASTA alignment of the core genome was generated using highly accurate PRANK, which is a probabilistic multiple alignment program (Löytynoja, 2014; Page et al., 2015). The number of core and unique genes among species of each genus was assessed from the Roary output and was used for the flower plots by computing R script in RStudio (R Core Team, 2022). A core gene phylogenetic tree was established using an ML tree inference tool Randomized Axelerated Maximum Likelihood – Next Generation (RAxML -NG) v0.8.0 (Kozlov et al., 2019), which combine the strengths of RAxML (Stamatakis, 2014) and Exascale Maximum Likelihood (ExaML) (Kozlov et al., 2015). The DNA substitution model, General Time Reversible (GTR) + GAMMA (G), was performed and ran separately with core genomes of type species of *Xanthomonas* spp. and *Stenotrophomonas* spp., with 1,000 bootstrap replicates. The core genome phylogenetic tree was displayed using a web-based tool Interactive Tree Of Life (iTOL v6, see text footnote 4) (Letunic and Bork, 2021). The Roary matrix with the presence and absence of core and accessory genes was combined with the core genome ML tree, and the results were visualized by conducting roary_plots.py (Page et al., 2015).

### 2.6 Antibiotic sensitivity assay

Antibiotic sensitivity assays were performed using disc diffusion methods described by Klair et al. (2022). Single colonies were picked from the pure culture plates of four new species strings and incubated in 10 mL of Luria-Bertani (LB) broth at 28°C with shaking at 200 rpm for 16 h. Light absorbance at 600 nm (OD600) of bacterial inoculum was adjusted to the value ~1.0, and 100 μL of inoculum was spread evenly on nutrient agar (NA, CRITERION^™^, Hardy Diagnostics). Seven antibiotics with different concentrations of bacitracin (50 mg/mL), chloramphenicol (50 mg/mL), gentamicin (50 mg/mL), kanamycin (50 mg/mL), penicillin (50 mg/mL), tetracycline (40 mg/mL), and polymyxin B sulfate (50 mg/mL) were tested. One Petri dish was divided into four zones, and three discs impregnated with each antibiotic solution and one disc soaked with sterile distilled water as control were placed in the center of each zone. Inhibition zones were observed and measured after incubating the plates at 28°C for 24 h.

## 3 Results

### 3.1 Genome assembly and annotation

**Figure 1:**
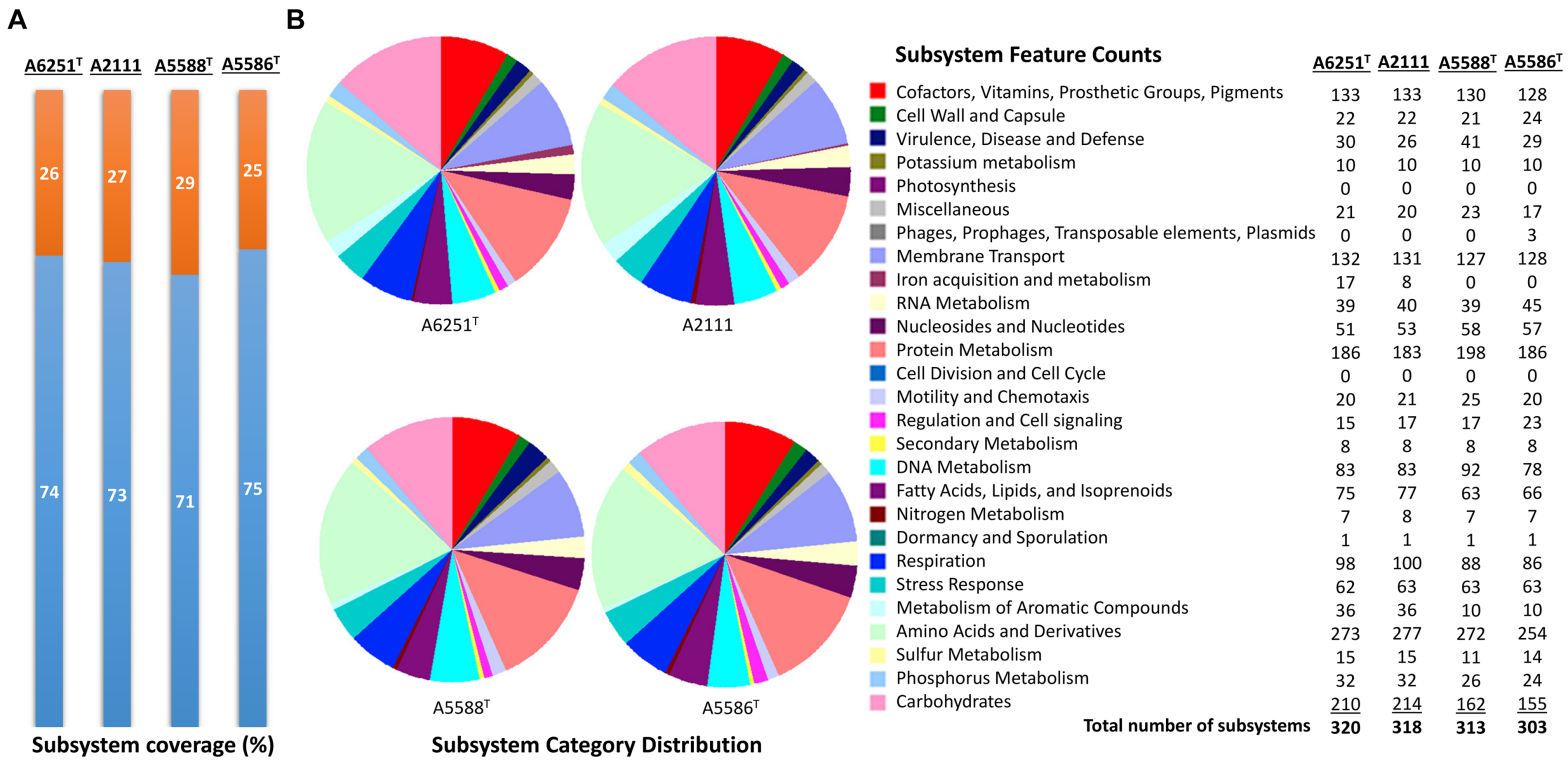
Subsystem annotation summary of new species strains, A6251^T^, A2111, A5588^T^, and A5586^T^ by conducting RAST web server. **(A)** The percentages of protein-coding genes present (orange portions) or absent (blue portions) in the RAST subsystem. **(B)** The pie chart and the number of subsystem features in total 27 categories found in four genomes of aroid strains.

The high-quality genomes of the strains A6251^T^, A5588^T^, and A5586^T^ were assembled using Unicycler v0.4.8, whereas strain A2111 had a better *de novo* assembly using another hybrid genome assembler, Flye v2.9.1 (Table 1). The genome sizes of new species strains from anthurium, A5588^T^ and A5586^T^, are 4.33 Mbp and 4.68 Mbp with 66.44 mol% and 65.3 mol% of GC content, respectively. In comparison, the GC content was higher in the other two strains, i.e., A6251^T^ (4.88 Mbp) from spathiphyllum and A2111 (4.87 Mbp) from colocasia, with 68.93 mol% and 68.88 mol% GC content, respectively (Table 1). Based on the annotation of NCBI-PGAPservice, the average CDS number of the four strains was 4,016. The strain A5588^T^ has the lowest CDS number, whereas the strain A5586^T^ has the highest number (Table 1). The CheckM completeness estimates were 99.9% in A6251^T^ and A2111 and 100% in A5588^T^ and A5586^T^ (Table 1). Although the CDS numbers estimated by RAST web server were slightly different from PGAP annotation, the strain A5588^T^ had the lowest CDS number which correlated with its genome size (data not shown). By contrast, the coverage of subsystem features presented in A5588^T^ was the highest and in A5586^T^ was the lowest (Figure 1A). Four strains comprised 23 out of the total 27 subsystem feature categories including virulence, stress response, membrane transport, DNA, and protein metabolism (Figure 1B). Notably, only strains A6251^T^ and A2111 contained proteins in the iron acquisition and metabolism subsystem but not strains A5588^T^ and A5586^T^ (Figure 1B). *Stenotrophomonas maltophilia* was reported to use two putative iron acquisition systems for the mediation of siderophores and heme as iron starvation (Kalidasan et al., 2018), implying that strains A5588^T^ and A5586^T^ from anthurium were different from the opportunistic human pathogen.

**Table 1:** Genome features of *Xanthomonas hawaiiensis* sp. nov., *Stenotrophomonas aracearum* sp. nov., and *S. oahuensis* sp. nov. in this study.

Species name	Genome size (Mbp)	No. of contigs	N50 (bp)	GC content (%)	CheckM complete-ness	CheckM contami-nation	No. of CDS^*^	Short read average coverage	Long read average coverage	Genome assembler	NCBI accession no.
*Xanthomonas hawaiiensis* A6251^T^	4.880993	1	4,880,993	68.93	99.9	0.9	4,087	203.00	20.75	Unicycler v0.4.8	CP115873
*Xanthomonas hawaiiensis* A2111	4.867870	3	4,828,414	68.88	99.9	0.8	4,085	184.95	21.43	Flye v2.9.1	JAQMHB000000000
*Stenotrophomonas aracearum* A5588^T^	4.328236	1	4,328,236	66.44	100	-	3,769	287.19	53.82	Unicycler v0.4.8	CP115543
*Stenotrophomonas oahuensis* A5586^T^	4.684619	2	4,623,839	65.30	100	-	4,121	243.04	43.90	Unicycler v0.4.8	CP115541-CP115542

^*^Indicates that the number of estimated CDS was generated from the NCBI PGAP pipeline.

### 3.2 Phylogenetic analyses

**Figure 2:**
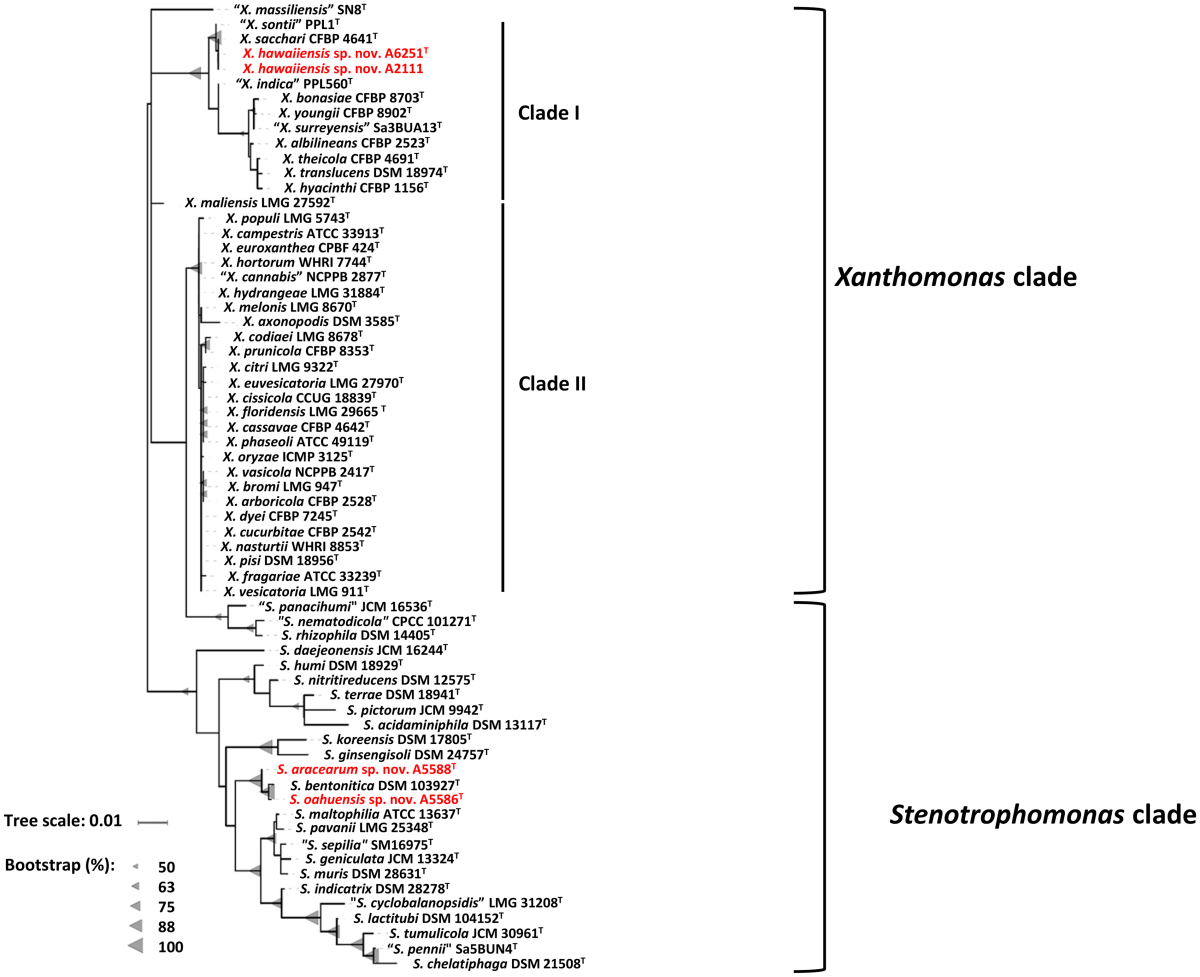
Maximum Likelihood phylogenetic tree based on almost full-length 16S rRNA gene sequences among three new species strains and type strains of *Xanthomonas* and *Stenotrophomonas* species. The tree scale bar indicates the number of nucleotide substitutions per sequence position. The range of gray triangles represents the degree of bootstrapping values.

The partial sequences of 16S rRNA gene were amplified using primer set P16S-F1 and P16S-R1 and deposited in the NCBI GenBank database under accession numbers OP962219 (A6251^T^), OP962220 (A2111), OP964727 (A5586^T^), and OP964728 (A5588^T^). The 16S rRNA gene sequences were retrieved from the whole genomes of new species strains, and 38 type strains of *Xanthomonas* species and 23 type strains of *Stenotrophomonas* species published in the NCBI database (Supplementary Table 1). The sequences of the nearly entire 16S rRNA gene ranging from 1,415 bp (*S. bentonitica* LMG 29893^T^) to 1,421 bp (*S. chelatiphaga* DSM 21508^T^) were analyzed for phylogenetic relationships. The 16S rRNA gene sequences of A6251^T^ and A2111 were identical with *X. sacchari* CFBP 4641^T^ and only one base was different from “*X. sontii”* PPL1^T^. A5588^T^ and A5586^T^ were closely related to each other and *S. bentonitica* LMG 29893^T^ and showed higher similarity values of 16S rRNA ranging from 99.6 to 99.8%. In the maximum likelihood (ML) phylogenetic tree, 16S rRNA gene sequences depicted better resolution within *Stenotrophomonas* species than *Xanthomonas* species because of very poor species discrimination, which was higher than the 98.7% cutoff of 16S similarity (Figure 2).

**Figure 3:**
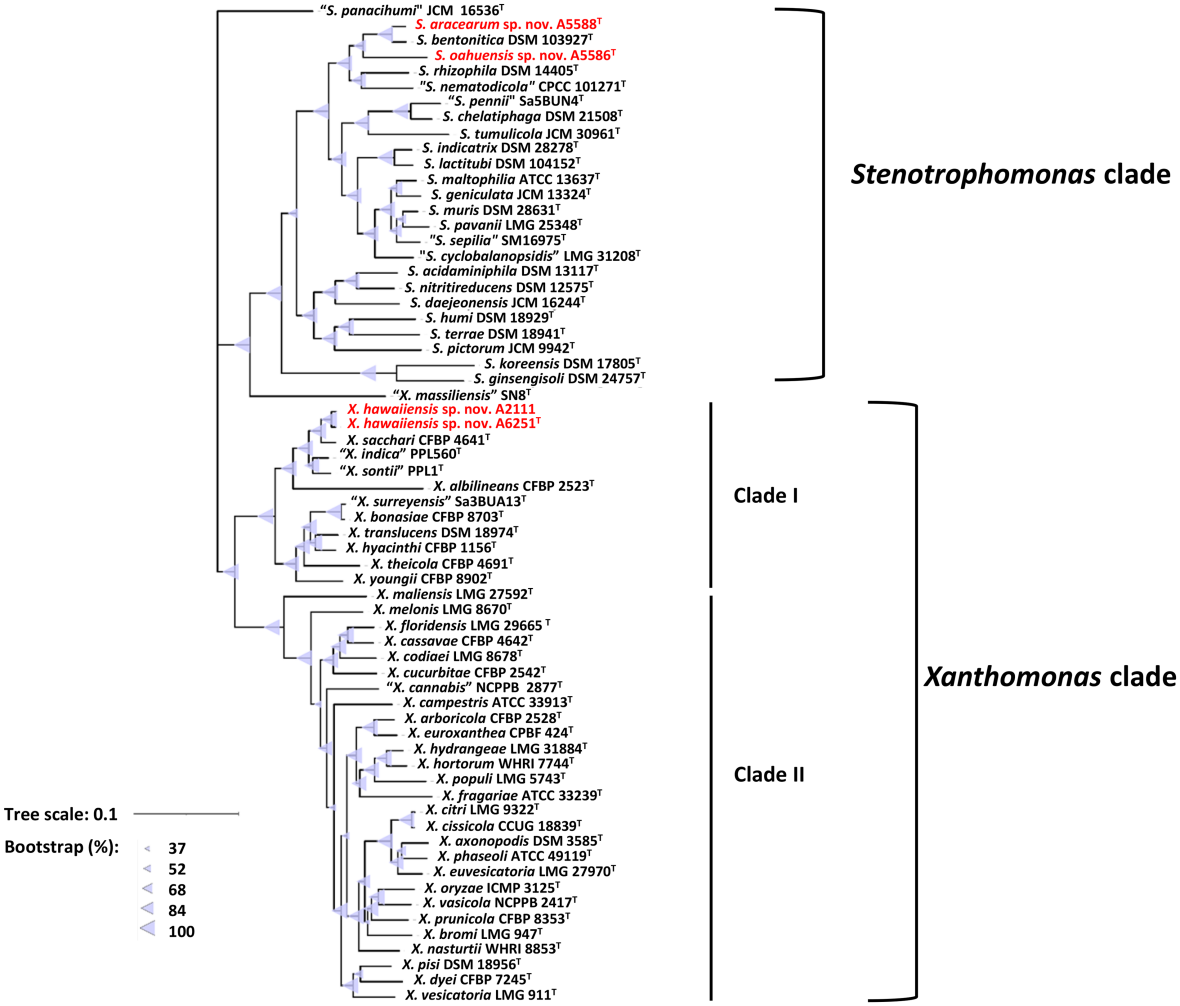
Maximum Likelihood phylogenetic tree based on concatenated sequence set of nine housekeeping genes, *atpD*, *dnaA*, *dnaK*, *gltA*, *gyrB*, *nuoD*, *ppsA*, *rpoH*, and *uvrB* of *Xanthomonas* and *Stenotrophomonas* species type strains. The scale bar represents the nucleotide substitutions per site. The range of purple triangles indicates the degree of bootstrapping support.

For more detailed phylogenetic analysis, nine housekeeping genes (*atpD*, *dnaA*, *dnaK*, *gltA*, *gyrB*, *nuoD*, *ppsA*, *rpoH*, and *uvrB*) were selected and retrieved from whole genomes of formerly mentioned type strains of *Xanthomonas* and *Stenotrophomonas* species. Total length of concatenated sequence with nine genes in alphabetic order was approximately 14.3 Kb, which contained the maximum ~2,443 bp of *gyrB* gene and the minimum ~879 bp of *uvrB* gene sequences. The similarity of the concatenated gene sequences of two strains A6251^T^ and A2111 was 99.3%; strain A5588^T^ and strain A5586^T^ showed 89.8% similarity. Based on nine housekeeping genes, the ML tree indicated that two major phylogenetic clades, Clade I and Clade II, were present within the *Xanthomonas* clade with high bootstrapping value support (Figure 3). Similar Clade I and II phylogenetic groupings were reported in the previous studies (Koebnik et al., 2021; Mafakheri et al., 2022; Rana et al., 2022). The strains A6251^T^ and A2111 formed a monoclade clustering with *X. sacchari*, *X. indica*, *X. sontii*, and *X. albilineans* in Clade I, which also include *X. surreyensis*, *X. bonasiae*, *X. traslucens*, *X. hyacinthi*, *X. theicola*, and *X. youngii* (Figure 3). *Stenotrophomonas bentonitica* consistently clustered with strains A5588^T^ and A5586^T^ with strong bootstrapping value. *Stenotrophomonas rhizophila* and *S. nematodicola* formed a clade closely related to the A5588^T^-A5586^T^-*S. bentonitica* clade (Figure 3); however, no grouping was formed in the 16S rRNA phylogenetic tree (Figure 2).

### 3.3 Overall genomic relatedness indices

To examine the accurate taxonomic classification, the overall genomic relatedness indices (OGRIs) including the values of ANI and dDDH of A6251^T^ from spathiphyllum and A2111 from colocasia were analyzed with other type strains of *Xanthomonas* species. Meanwhile, A5588^T^ and A5586^T^ strains were compared with other type strains in *Stenotrophomonas*. The general cutoff values of ANI and dDDH for species delineation are lower than 95–96 and 70%, respectively (Goris et al., 2007; Richter and Rosselló-Móra, 2009; Meier-Kolthoff et al., 2013). Strains A6251^T^ and A2111 shared 98.4% ANI and 85.2% dDDH with each other, which indicated that two strains belong to the same species. Based on the pairwise comparisons of the other *Xanthomonas* spp. reference genomes with either A6251^T^ or A2111, the ANI and dDDH values were 83.4–94.9% and 22.3–59.3%, respectively, which strongly signified that A6251^T^ and A2111 are distinguished from the others and should be considered a novel lineage (Table 2). Despite that *X. sacchari* CFBP 4641^T^ shared slightly higher OrthoANI values (95.04, 95.1) with A6251^T^ and A2111, other OGRIs supported the assignment as a new species (Table 2). The estimations of ANI and dDDH of anthurium strains, A5588^T^ and A5586^T^, were 86.4 and 28.2%, respectively. Both strains shared ANI and dDDH values lower than 90% (83.4–86.8%) and 30% (20.7–29.9%) with other type strains of *Stenotrophomonas* spp., respectively, except for that A5588^T^ and *S. bentonitica* LMG 29893^T^ shared 94.7% of ANI and 56.4% of dDDH sequence identities (Table 3). In addition to ANI and dDDH values, other OGRIs including AP, OrthoANI, and G + C differences supported that A5588^T^ and A5586^T^ are two novel species (Table 3). To combine the phylogenetic analyses and evidence of OGRIs, three novel species were proposed, i.e., *X. hawaiiensis* sp. nov. strains A6251^T^ and A2111; *S. aracearum* sp. nov. strain A5588^T^; and, *S. oahuensis* sp. nov. strain A5586^T^.

**Table 2:** Overall genomic relatedness indices (OGRIs) comparison of new species, *Xanthomonas hawaiienesis* sp. nov., strains with other type strains of *Xanthomonas* species.

	AP (%)		ANI (%)		OrthoANI (%)		dDDH (%)		G + C difference (%)	
Species name	A6251^T^	A2111	A6251^T^	A2111	A6251^T^	A2111	A6251^T^	A2111	A6251^T^	A2111
*Xanthomonas hawaiiensis* sp. nov. A6251^T^	100	95.2	100	98.4	100	98.39	100	85.2	0.0	0.05
*Xanthomonas hawaiiensis* sp. nov. A2111	95.2	100	98.4	100	98.39	100	85.2	100	0.05	0.0
*Xanthomonas albilineans* CFBP 2523^T^	48.6	48.4	85.4	85.4	84.62	84.52	28.4	28.5	5.87	5.82
*Xanthomonas arboricola* CFBP 2528^T^	22.8	22.9	84.1	84.2	79.82	79.83	23.4	23.4	3.47	3.42
*Xanthomonas axonopodis* DSM 3585^T^	19.5	19.6	83.7	83.8	79.02	78.95	23.0	23.0	4.45	4.4
*Xanthomonas bonasiae* CFBP 8703^T^	60.8	61.1	87.8	87.8	87.45	87.46	32.6	32.7	0.13	0.08
*Xanthomonas bromi* LMG 947^T^	19.4	19.8	83.8	83.7	79.01	78.90	22.9	23.0	4.87	4.82
*Xanthomonas campestris* ATCC 33913^T^	21.1	21.1	84.0	84.0	79.33	79.23	23.1	23.1	3.86	3.81
“*Xanthomonas cannabis*” NCPPB 2877^T^	22.2	22.6	84.0	83.9	79.59	79.51	23.4	23.4	3.15	3.1
*Xanthomonas cassavae* CFBP 4642^T^	21.1	21.2	84.1	84.1	79.63	79.53	23.4	23.4	3.7	3.65
*Xanthomonas cissicola* CCUG 18839^T^	19.9	20.0	83.9	83.8	79.14	79.06	22.9	22.9	4.54	4.49
*Xanthomonas citri* LMG 9322^T^	19.9	20.1	83.9	83.9	78.95	78.97	22.8	22.9	4.28	4.24
*Xanthomonas codiaei* LMG 8678^T^	23.1	22.8	84.2	84.2	79.87	79.79	23.6	23.6	2.89	2.85
*Xanthomonas cucurbitae* CFBP 2542^T^	21.9	22.1	84.1	84.2	79.51	79.62	23.1	23.2	3.49	3.44
*Xanthomonas dyei* CFBP 7245^T^	19.0	19.2	83.8	83.8	79.17	79.08	22.9	23.0	4.65	4.6
*Xanthomonas euroxanthea* CPBF 424^T^	23.4	23.7	84.2	84.2	79.90	79.88	23.5	23.5	3.04	2.99
*Xanthomonas euvesicatoria* LMG 27970^T^	20.6	20.7	83.9	84.0	79.12	79.14	23.6	23.6	4.25	4.2
*Xanthomonas floridensis* LMG 29665^T^	21.3	21.4	84.1	84.1	79.51	79.49	23.4	23.4	3.56	3.51
*Xanthomonas fragariae* ATCC 33239^T^	15.7	15.9	83.4	83.4	78.68	78.6	22.3	22.5	6.71	6.66
*Xanthomonas hortorum* WHRI 7744^T^	19.0	19.2	83.8	83.8	79.01	78.93	22.9	22.9	5.31	5.26
*Xanthomonas hyacinthi* CFBP 1156^T^	55.8	56.3	87.9	87.8	87.82	87.65	33.7	33.7	0.9	0.85
*Xanthomonas hydrangeae* LMG 31884^T^	19.6	19.7	84.0	83.9	79.11	79.16	23.1	23.1	5.33	5.28
“*Xanthomonas indica*” PPL560^T^	84.2	84.1	93.3	93.3	93.48	93.38	50.7	50.8	0.54	0.59
*Xanthomonas maliensis* LMG 27592^T^	22.5	22.4	84.3	84.4	79.55	79.50	23.0	23.1	2.75	2.7
“*Xanthomonas massiliensis* “SN8^T^	21.4	21.6	84.2	84.2	80.39	80.46	23.4	23.4	1.6	1.64
*Xanthomonas melonis* LMG 8670^T^	22.5	22.6	84.1	84.1	79.67	79.62	23.3	23.4	2.82	2.77
*Xanthomonas nasturtii* WHRI 8853^T^	21.0	20.8	84.1	83.7	79.36	79.26	23.2	23.1	4.46	4.41
*Xanthomonas oryzae* ICMP 3125^T^	17.4	17.5	83.7	83.7	78.94	78.88	22.9	22.8	5.24	5.19
*Xanthomonas phaseoli* ATCC 49119^T^	20.0	20.2	84.0	84.0	79.16	79.23	23.1	23.0	4.11	4.06
*Xanthomonas pisi* DSM 18956^T^	18.3	18.3	84.0	84.0	79.18	79.18	23.0	23.1	4.21	4.16
*Xanthomonas populi* LMG 5743^T^	17.9	18.1	83.6	83.6	78.79	78.61	22.6	22.5	5.62	5.57
*Xanthomonas prunicola* CFBP 8353^T^	19.1	19.2	84.0	83.9	78.86	78.91	22.9	22.9	4.96	4.91
*Xanthomonas sacchari* CFBP 4641^T^	86.5	86.2	94.9	94.9	95.04	95.10	59.3	59.3	0.13	0.18
“*Xanthomonas sontii* “PPL1^T^	80.2	81.1	93.8	93.8	94.11	94.16	53.9	53.9	0.06	0.1
“*Xanthomonas surreyensis* “Sa3BUA13^T^	63.9	63.9	87.9	87.9	87.45	87.44	32.6	32.7	0.12	0.07
*Xanthomonas theicola* CFBP 4691^T^	50.7	50.9	87.6	87.6	87.04	86.91	32.3	32.4	0.76	0.71
*Xanthomonas translucens* DSM 18974^T^	56.7	56.8	87.4	87.4	87.16	87.04	32.3	32.3	1.21	1.16
*Xanthomonas vasicola* NCPPB 2417^T^	17.9	18.1	83.6	83.7	78.56	78.5	22.8	22.8	5.61	5.56
*Xanthomonas vesicatoria* LMG 911^T^	18.8	18.7	83.9	83.9	78.88	78.77	22.8	22.6	4.87	4.82
*Xanthomonas youngii* CFBP 8902^T^	49.1	49.3	87.1	87.1	86.13	86.13	30.8	30.8	0.87	0.92

The species name between two ditto marks (“) indicates the invalidly published species. Alignment Percentage (AP) and Average Nucleotide Identity (ANI) values were calculated using CLC Genomics Workbench 22.0.2; Average Nucleotide Identity by Orthology (OrthoANI) values were estimated using Orthologous Average Nucleotide Identity tool (OAT); digital DNA–DNA Hybridization (dDDH) and the differences of G + C content (mol%) were inferred on Type Strain Genome Server (TYGS) web server.

**Table 3:** Overall genomic relatedness indices (OGRIs) comparison of new species, *Stenotrophomonas oahuensis* sp. nov. and *S. aracearum* sp. nov. within other species in the genus.

	AP (%)		ANI (%)		OrthoANI (%)		dDDH (%)		G + C difference (%)	
Species name	A5586^T^	A5588^T^	A5586^T^	A5588^T^	A5586^T^	A5588^T^	A5586^T^	A5588^T^	A5586^T^	A5588^T^
*Stenotrophomonas oahuensis* sp. nov. A5586^T^	100	53.9	100	86.4	100	84.41	100	28.2	0.0	1.15
*Stenotrophomonas aracearum* sp. nov. A5588^T^	53.9	100	86.4	100	84.41	100	28.2	100	1.15	0.0
*Stenotrophomonas acidaminiphila* DSM 13117^T^	22.0	26.0	84.1	84.3	79.62	80.67	23.1	23.6	3.61	2.46
*Stenotrophomonas bentonitica* DSM 103927^T^	52.9	80.7	86.6	94.7	84.44	94.35	28.4	56.4	1.17	0.02
*Stenotrophomonas chelatiphaga* DSM 21508^T^	31.1	35.0	84.5	84.7	80.60	81.29	23.8	24.2	1.54	0.40
*“Stenotrophomonas cyclobalanopsidis”* LMG 31208^T^	23.5	27.6	84.8	85.0	81.47	82.17	24.5	25.2	1.84	0.69
*Stenotrophomonas daejeonensis* JCM 16244^T^	31.0	34.8	84.0	84.3	80.02	80.97	23.5	24.1	3.27	2.12
*Stenotrophomonas geniculata* JCM 13324^T^	10.9	12.1	84.8	85.2	81.05	81.78	24.5	24.9	0.89	0.26
*Stenotrophomonas ginsengisoli* DSM 24757^T^	18.1	20.2	83.6	83.4	76.30	77.01	20.7	20.9	0.59	0.56
*Stenotrophomonas humi* DSM 18929^T^	32.7	35.5	83.7	84.0	78.35	79.09	22.6	22.7	1.25	2.4
*Stenotrophomonas indicatrix* DSM 28278^T^	10.6	11.7	84.7	85.0	80.84	81.55	24.3	24.9	1.12	0.03
*Stenotrophomonas koreensis* DSM 17805^T^	31.0	33.7	83.6	83.6	76.47	76.76	20.7	20.7	0.81	0.34
*Stenotrophomonas lactitubi* DSM 104152^T^	30.5	34.2	84.8	85.0	80.92	81.58	24.3	24.6	0.58	0.57
*Stenotrophomonas maltophilia* ATCC 13637^T^	21.7	25.1	84.9	85.2	81.11	81.72	24.7	25.0	0.88	0.27
*“Stenotrophomonas muris* DSM 28631^T^	33.2	37.0	84.9	85.1	81.24	81.8	24.6	25.0	1.39	0.24
*“Stenotrophomonas nematodicola”* CPCC 101271^T^	20.6	23.7	85.6	86.5	83.42	84.96	26.8	28.6	2.03	0.89
*Stenotrophomonas nitritireducens* DSM 12575^T^	46.4	53.7	84.1	84.5	79.74	80.75	23.4	24.1	3.05	1.90
*“Stenotrophomonas panacihumi”* JCM 16536^T^	18.0	20.4	83.9	84.1	78.44	79.17	22.2	22.5	3.55	2.40
*Stenotrophomonas pavanii* LMG 25348^T^	27.3	30.4	84.9	85.1	81.24	81.74	24.4	24.9	1.94	0.79
*“Stenotrophomonas pennii”* Sa5BUN4^T^	35.1	39.1	84.5	84.6	80.35	80.88	23.5	23.8	1.15	0.00
*Stenotrophomonas pictorum* JCM 9942^T^	31.9	35.5	83.9	84.3	79.24	79.87	22.5	22.9	0.72	0.43
*Stenotrophomonas rhizophila* DSM 14405^T^	44.7	51.4	85.9	86.8	84.06	85.61	27.7	29.9	2.00	0.86
*“Stenotrophomonas sepilia”* SM16975^T^	19.5	23.1	84.8	85.1	81.02	81.48	24.4	24.8	1.15	0.00
*Stenotrophomonas terrae* DSM 18941^T^	31.0	34.6	83.8	83.9	78.59	79.03	22.3	22.7	1.41	2.56
*Stenotrophomonas tumulicola* JCM 30961^T^	31.9	34.9	84.4	84.9	79.97	80.87	23.5	23.9	0.31	0.84

The species name between two ditto marks (“) indicates the invalidly published species. Alignment Percentage (AP) and Average Nucleotide Identity (ANI) values were calculated using CLC Genomics Workbench 22.0.2; Average Nucleotide Identity by Orthology (OrthoANI) values were estimated using Orthologous Average Nucleotide Identity tool (OAT); digital DNA–DNA Hybridization (dDDH) and the differences of G + C content (mol%) were inferred on Type Strain Genome Server (TYGS) web server.

### 3.4 Pan- and core-genomic analyses

**Figure 4:**
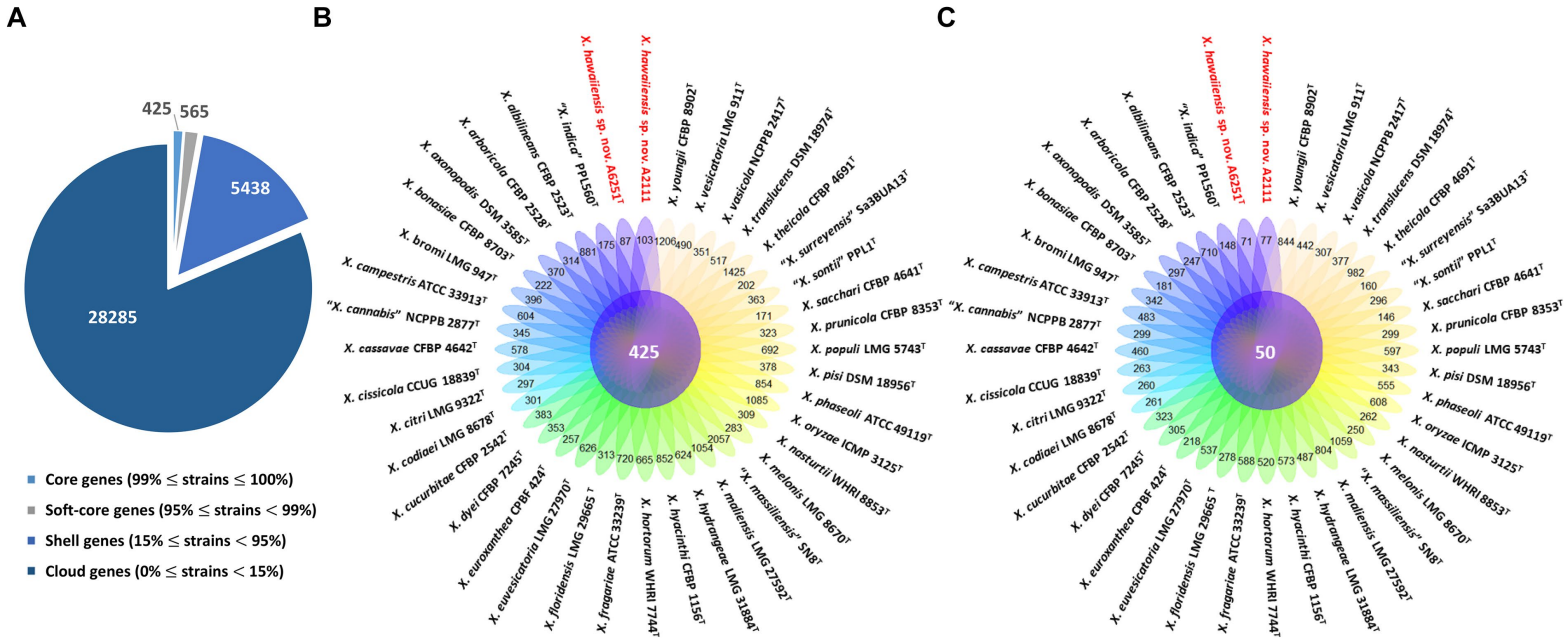
Pan-genome analyses of *Xanthomonas hawaiienses* sp. nov. (A6251^T^ and A2111) with other type strains of *Xanthomonas* species. **(A)** Numbers of core, soft-core, shell, and cloud genes within 40 genomes of type strains of *Xanthomonas* species. **(B)** Floral plot showing the number of core orthologous genes in the center and the number of unique genes on each petal. **(C)** The number of common hypothetical protein encoding genes in the center of floral plot and the number of unique hypothetical protein encoding genes of each *Xanthomonas* strain on each petal.

**Figure 5:**
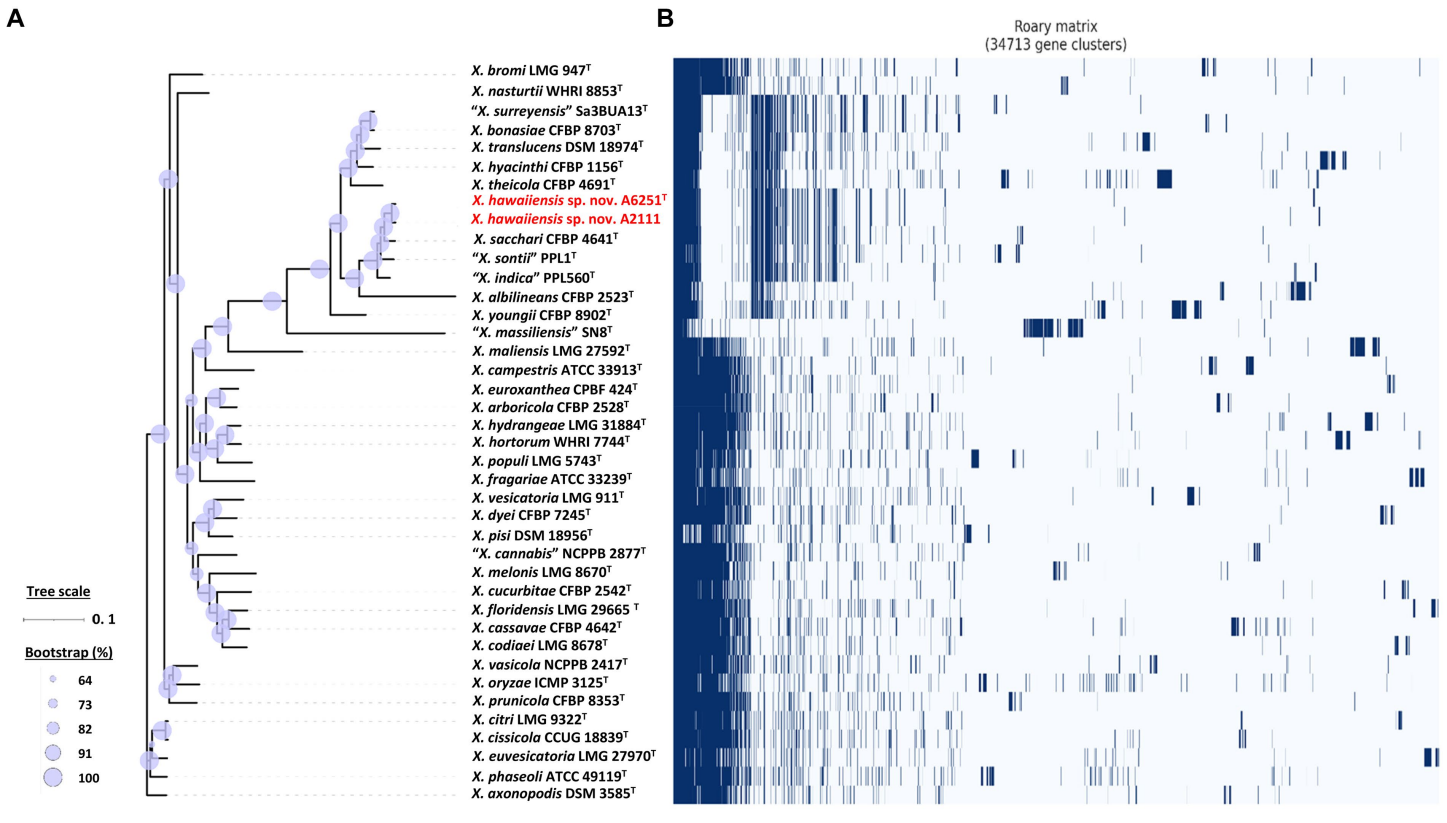
Core- and pan-genome analyses of 40 *Xanthomonas* species including new species strains. **(A)** Core genome-based ML phylogenetic tree of *Xanthomonas hawaiienses* sp. nov. (A6251^T^ and A2111) with other type strains of *Xanthomonas* species. Tree scale bar represents the nucleotide substitutions per site. The range of purple circles indicates the percentage of bootstrapping confidence. **(B)** Pan genome-based Roary matrix of the presence and absence of genes among 40 coordinated *Xanthomonas* species. Dark blue blocks represent genes and pale blue blocks are missing genes in the genomes.

Among 40 reference genomes of *Xanthomonas* spp. including *X. hawaiiensis* sp. nov. strains A6251^T^ and A2111, 425 core orthologous genes (99% ≤ strains ≤100%) and 28,285 cloud genes (0% ≤ strains <15%) were found (Figure 4A). The lowest two numbers of unique genes present in A6251^T^ and A2111 were 87 and 103, respectively, and follow *X. sacchari* CFBP 4641^T^ which had 171 unique genes, as shown in the Figure 4B. The number of exclusive hypothetical protein encoded genes was comparatively lower in strains A6251^T^ and A2111, whereas 50 common hypothetical proteins existed in all type strains of *Xanthomonas* spp. (Figure 4C). Based on the phylogenetic tree constituted with 425 core genes, the closest relative of *X. hawaiiensis* sp. nov. was *X. sacchari* CFBP 4641^T^, which successively clustered with *“X. sontii”* PPL1^T^ and “*X. indica*” CFBP 9039^T^ in *Xanthomonas* clade I species (Figure 5). The groupings were concordant with the previously described MLSA tree (Figure 3). The 34,713 gene clusters estimated in the Roary matrix revealed that the genomes of xanthomonads were highly diversified (Figure 5).

**Figure 6:**
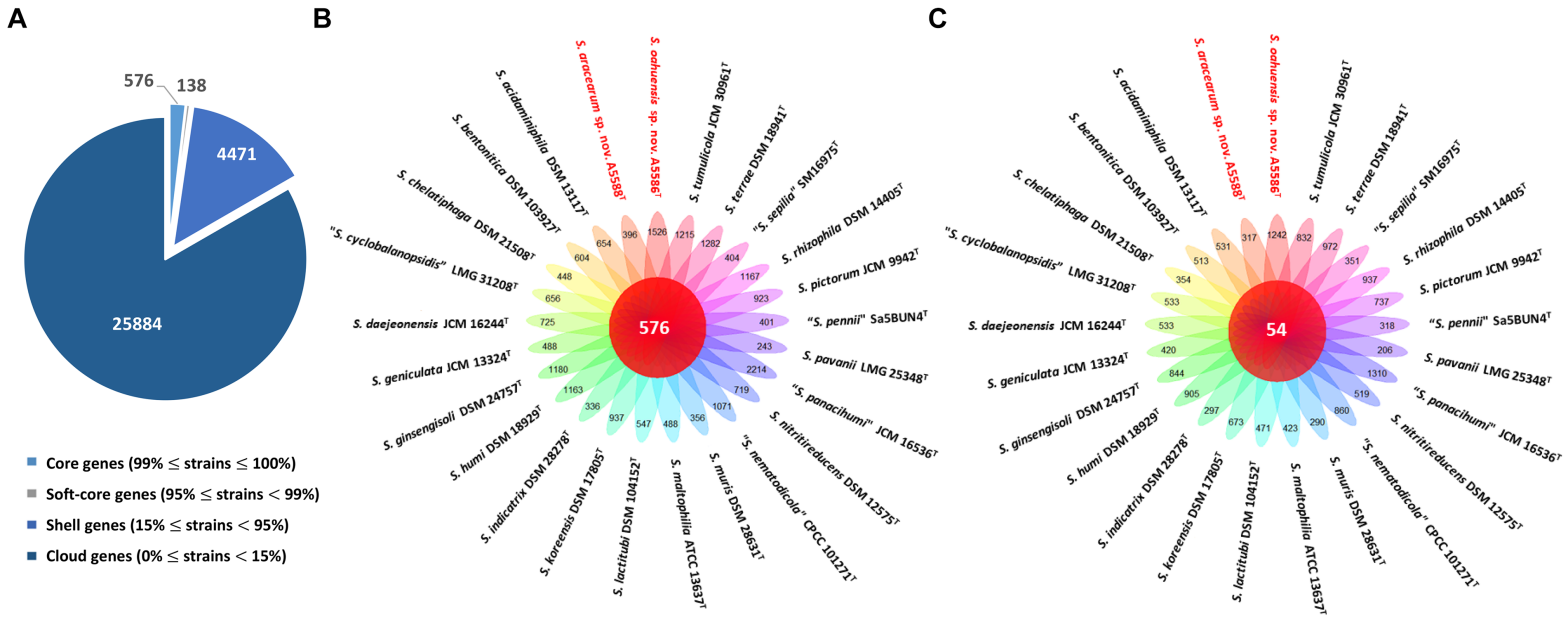
Pan-genome analyses of *Stenotrophomonas aracearum* sp. nov. (A5588^T^) and *S. oahuensis* sp. nov. (A5586^T^) with other type strains of *Stenotrophomonas* species. **(A)** Numbers of core, soft-core, shell, and cloud genes within 25 genomes of type strains of *Stenotrophomonas* species. **(B)** Floral plot showing the number of core orthologous genes in the center and the number of unique genes on each petal. **(C)** The number of common hypothetical protein encoding genes in the center of floral plot and the number of unique hypothetical protein encoding genes of each *Stenotrophomonas* species on each petal.

**Figure 7:**
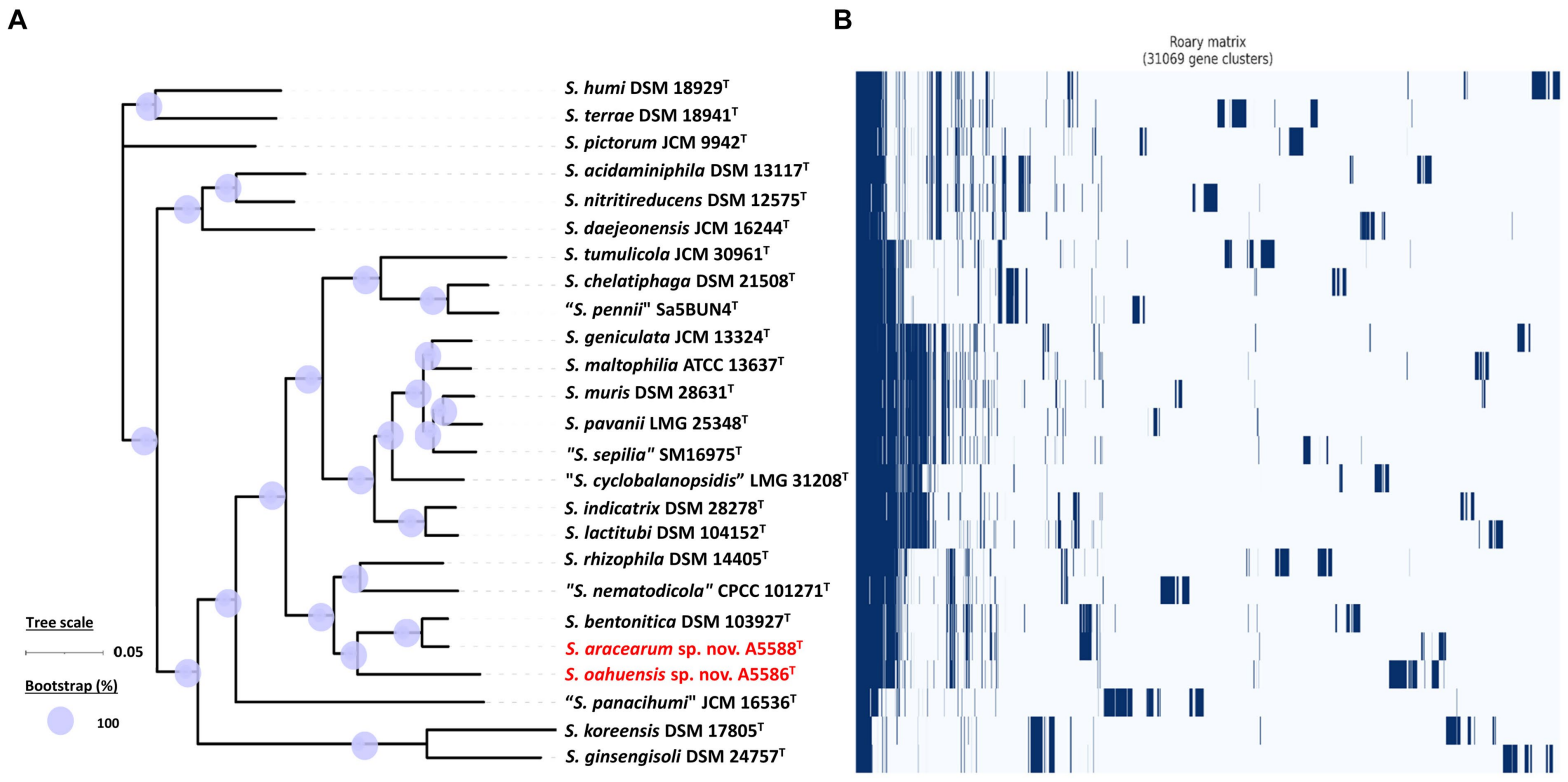
Core- and pan-genome analyses of 25 *Stenotrophomonas* species including two new species type strains. **(A)** Core genome-based ML phylogenetic tree of *S. aracearum* sp. nov. (A5588^T^) and *S. oahuensis* sp. nov. (A5586^T^) along with other type strains of *Stenotrophomonas* species. Tree scale bar represents the substitutions per nucleotide position. The purple circles represent 100% bootstrapping support. **(B)** Pan genome-based Roary matrix of the presence and absence of genes among all coordinated *Stenotrophomonas* species. Dark blue blocks indicate genes present and pale blue blocks indicate genes absent in the genomes.

On the other hand, the pan genome size of 20 *Stenotrophomonas* spp. type strains, including *S. aracearum* sp. nov. (A5588^T^) and *S. oahuensis* sp. nov. (A5586^T^), was 31,069 with 576 core genes (Figures 6A, 7). The genome of the strain A5588^T^ contained 396 unique genes, 317 of which were hypothetical protein encoding genes; whereas, a high number of hypothetical protein encoding genes (1,242 genes) were harbored in the genome of the strain A5586^T^, possessing total 1,526 unique genes (Figures 6B–C). As presented in the 9-gene ML tree (Figure 3), A5588^T^ and *S. bentonitica* DSM 103927^T^ were closely clustered together and grouped with A5586^T^, which was a sister group of the clade formed with *S. rhizophila* DSM 14405^T^ and *“S. nematodicola”* CPCC 101271^T^ (Figure 7). The average number of unique genes with unknown functions was higher in 25 *Stenotrophomonas* spp. than 40 *Xanthomonas* spp. (806 > 538), implying higher genetic diversity within stenotrophomonads, which warrants further investigations on *Stenotrophomonas* species.

### 3.5 Antibiotic sensitivity assays

The inhibition zones with seven tested antibiotics, namely, bacitracin (50 mg/mL), chloramphenicol (50 mg/mL), gentamicin (50 mg/mL), kanamycin (50 mg/mL), penicillin (50 mg/mL), tetracycline (40 mg/mL), and polymyxin B sulfate (50 mg/mL), indicated various degrees of sensitivity of four new species strains. Strains A6251^T^, A2111, and A5586^T^ were sensitive to all tested antibiotics, whereas strain A5588^T^ was sensitive to all tested antibiotics except penicillin (Supplementary Table 2). Strains A6251^T^ and A2111, belonging to the same new species, displayed similar results, except for the tolerance to polymyxin B sulfate. Notably, A5586^T^ displayed a very small inhibition zone (0.1 cm in radius) surrounding the discs of bacitracin on the NA plate after incubating at 28°C for 24 h (Supplementary Table 2).

## 4 Descriptions of new species

### 4.1 *Xanthomonas hawaiiensis* sp. nov. (ha.waii.en′sis. N.L. fem. adj. *hawaiiensis*, of or belonging to Hawaii, a state of the United States, referring to the geographical origin of the new species)

Colonies of the type strain A6251^T^ are yellow (Honey, Hex code #FFC30B), circular shape, mucoid consistency, smooth surface, convex relief with entire margins, and 0.3–0.6 (avg. 0.45) mm in diameter on yeast dextrose calcium carbonate (YDC) medium plate after incubating at 28°C for 2 days. Cells are gram-negative and able to utilize dextrin, D-maltose, D-trehalose, D-cellobiose, gentiobiose, sucrose, D-turanose, α-D-lactose, D-melibiose, Β-methyl-D-glucoside, D-salicin, N-acetyl-D-glucosamine, α-D-glucose, D-mannose, D-fructose, D-galactose, L-fucose, 1% NaCl, 1% sodium lactate, glycerol, gelatin, L-glutamic acid, lincomycin, pectin, quinic acid, vancomycin, tetrazolium violet, tetrazolium blue, citric acid, bromo-succinic acid, lithium chloride, Tween 40, and acetic Acid. In contrast, cells are unable to oxidize stachyose, D-raffinose, N-acetyl-β-D-mannosamine, N-acetyl-D-galactosamine, N-acetyl-neuraminic acid, 8% NaCl, inosine, fusidic acid, D-sorbitol, D-mannitol, D-arabitol, myo-inositol, D-aspartic acid, minocycline, L-arginine, L-histidine, L-pyroglutamic acid, guanidine HCl, D-gluconic acid, mucic acid, D-saccharic acid, p-hydroxy-phenylacetic acid, D-lactic acid methyl ester, α-keto-glutaric acid, D-malic acid, γ-aminobutryric acid, α-hydroxybutyric acid, α-ketobutyric acid, formic acid, sodium butyrate, and sodium bromate. Some utilization of carbon resources and chemical components showed borderline results or inconsistency between two strains after growing cell suspension in GEN III Microplate (Biolog Inc., Hayward, CA, USA) at 28°C for 24 h.

*X. hawaiiensis* sp. nov. is sensitive to seven tested antibiotics, including bacitracin (50 mg/mL), chloramphenicol (50 mg/mL), gentamicin (50 mg/mL), kanamycin (50 mg/mL), penicillin (50 mg/mL), tetracycline (40 mg/mL), and polymyxin B sulfate (50 mg/mL). The genome size of type strain A6251^T^ is 4.88 Mbp with 68.93 mol% of DNA G + C content.

The type strain A6251^T^ = D-93^T^ = ICMP 25022^T^ = LMG 33200^T^ was isolated from *Spathiphyllum* (Araceae family) in 1985 in Hawaii, USA. Another strain A2111 = D-194 = ICMP 25023 = LMG 33199 was isolated from *Colocasia* (Araceae family) in 1986 in Hawaii, USA.

### 4.2 *Stenotrophomonas aracearum* sp. nov. (a.ra.ce.a’rum. N.L. gen. fem. pl. n. *aracearum*, representative of plants belonging to the Araceae family)

Colonies of *S. aracearum* strain A5588^T^ are dark yellow (Mustard, Hex code #E8B828), irregular shape, butyrous consistency, smooth surface, raised relief with entire margins, and 0.4–0.5 mm (average 0.45) in diameter on YDC medium plates after incubation at 28°C for 2 days. Cells are gram-negative and able to utilize D-maltose, D-cellobiose, gentiobiose, N-acetyl-D-glucosamine, N-acetyl-D-galactosamine, α-D-glucose, D-mannose, 1% sodium lactate, D-serine, troleandomycin, rifamycin SV, gelatin, lincomycin, guanidine HCl, vancomycin, tetrazolium violet, tetrazolium Blue, α-ketoglutaric acid, L-malic acid, bromo-succinic acid, acetic acid, and aztreonam. Cells grow under pH 6 and 1% NaCl but neither at pH 5 nor 8% NaCl. In the contrary, cells are unable to oxidize sucrose, D-turanose, stachyose, D-raffinose, α-D-lactose, D-melibiose, Β-methyl-D-glucoside, N-acetyl-β-D-mannosamine, N-acetyl-neuraminic acid, D-galactose, 3-methyl-glucose, inosine, fusidic acid, D-sorbitol, D-mannitol, D-arabitol, myo-inositol, glycerol, D-glucose-6-PO4, D-aspartic acid, D-serine, minocycline, L-arginine, L-aspartic acid, L- glutamic acid, L-histidine, L-pyroglutamic acid, L-serine, pectin, D-galacturonic acid, D-gluconic acid, mucic acid, quinic acid, D-saccharic acid, p-hydroxy-phenylacetic acid, D-lactic acid methyl ester, L-lactic acid, citric acid, D-malic acid, nalidixic acid, potassium tellurite, γ-aminobutryric acid, α-hydroxybutyric acid, α-ketobutyric acid, β-hydroxy-D, L-butyric acid, acetoacetic acid, formic acid, sodium butyrate, and sodium bromate. Some utilization of carbon sources and chemical components, such as dextrin and glucuronamide showed faded positive results after growing A5588^T^ cell suspension in GEN III Microplate (Biolog Inc., Hayward, CA, USA) at 28°C for 24 h.

*S. aracearum* sp. nov. was sensitive to six tested antibiotics, namely, bacitracin (50 mg/mL), chloramphenicol (50 mg/mL), gentamicin (50 mg/mL), kanamycin (50 mg/mL), tetracycline (40 mg/mL), and polymyxin B sulfate (50 mg/mL) but resistant to penicillin (50 mg/mL) on NA plates. The genome size of type strain A5588^T^ is 4.33 Mbp with 66.44 mol% of DNA G + C content.

The type strain A5588^T^ = D-61-1L^T^ = ICMP 25025^T^ = LMG 33202^T^ was isolated from *Anthurium* (Araceae family) in 1985 in Hawaii, USA.

### 4.3 *Stenotrophomonas oahuensis* sp. nov. (o.a.hu.en’sis. N.L. fem. adj. *oahuensis*, of or belonging to the island of Oahu in Hawaii, referring to the geographical origin of the new species)

Colonies of the *S. oahuensis* strain A5586^T^ are dark yellow (Butterscotch, Hex code #FABD02), circular shape, butyrous consistency, smooth surface, flat relief with undulate margins, and 0.4–0.7 mm (average 0.55) in diameter on YDC medium plates after incubation at 28°C for 2 days. Cells are gram-negative and able to utilize dextrin, D-maltose, D-trehalose, D-cellobiose, gentiobiose, Β-methyl-D-glucoside, D-salicin, N-acetyl-D-glucosamine, α-D-glucose, D-mannose, 1% sodium lactate, gelatin, glycyl-L-proline, lincomycin, guanidine HCl, vancomycin, tetrazolium violet, tetrazolium blue, citric acid, α-ketoglutaric acid, L-malic acid, bromo-succinic acid, lithium chloride, propionic acid, acetic acid, and aztreonam. Cells grow under the conditions of pH 6, 1% NaCl, or 4% NaCl but cells survive neither pH 5 nor 8% NaCl solution. In contrast, cells are unable to oxidize sucrose, stachyose, D-raffinose, N-acetyl-β-D-mannosamine, N-acetyl-neuraminic acid, D-galactose, 3-methyl-glucose, inosine, D-fucose, L-fucose, L-rhamnose, inosine, fusidic acid, D-sorbitol, D-mannitol, D-arabitol, myo-inositol, glycerol, D-glucose-6-PO4, D-aspartic acid, D-serine, rifamycin SV, minocycline, L-arginine, L-aspartic acid, L- glutamic acid, L-histidine, L-pyroglutamic acid, L-serine, pectin, D-galacturonic acid, D-gluconic acid, D-glucuronic acid, mucic acid, quinic acid, D-saccharic acid, p-hydroxy-phenylacetic acid, D-lactic acid methyl ester, L-lactic acid, D-malic acid, nalidixic acid, potassium tellurite, γ-amino-butryric acid, α-hydroxy-butyric acid, β-hydroxy-D, L-butyric acid, α-keto-butyric acid, acetoacetic acid, formic acid, and sodium bromate. Some utilization of carbon resources and chemical components, such as D-turanose and sodium butyrate, showed faded positive results after growing A5586^T^ cell suspension in GEN III Microplate (Biolog Inc., Hayward, CA, USA) at 28°C for 24 h.

*S. oahuensis* sp. nov. was sensitive to seven tested antibiotics, namely, bacitracin (50 mg/mL), chloramphenicol (50 mg/mL), gentamicin (50 mg/mL), kanamycin (50 mg/mL), penicillin (50 mg/mL), tetracycline (40 mg/mL), and polymyxin B sulfate (50 mg/mL). The genome size of type strain A5586^T^ is 4.68 Mbp, which includes a chromosome (4.62 Mbp) and a plasmid (60.78 Kbp). The DNA G + C content of the type strain is 65.3 mol%.

The type strain A5586^T^ = D-31^T^ = ICMP 25024^T^ = LMG 33201^T^ was isolated from *Anthurium* (Araceae family) in 1981 in Hawaii, USA.

## 5 Discussion

The genera *Xanthomonas* and *Stenotrophomonas* are phylogenetically and evolutionarily linked and are also found frequently together in several niches, including environmental reservoirs (plants and soil) and biofilters used for waste gas treatment of animal-rendering plants (Lipski and Altendorf, 1997; Finkmann et al., 2000; Ryan et al., 2009). Although more studies are focused on phyto- and human-pathogenic species, the versatility of *Xanthomonas* and *Stenotrophomonas* spp. has the potential to be applied to many different fields and needs to be explored further.

The well-known industrial biopolymer, which is also a food additive, is xanthan gum produced by *X. campestris* and other *Xanthomonas* species (Margaritis and Zajic, 1978; Kennedy and Bradshaw, 1984; Gumus et al., 2010). Production of other bioactive secondary metabolites from xanthomonads include the siderophore xanthoferrin, which acts as a bioproduction agent under low iron conditions (Pandey et al., 2017), and the pigment xanthomonadin, analogs of which have antioxidant potential (Madden et al., 2019). The subsystem features of iron acquisition and metabolism based on RAST annotation webserver (Figure 1B) suggest that *X. hawaiiensis* sp. nov. strains, A6251^T^ and A2111, are capable of surviving inside the hosts (Expert et al., 1996). The *xss* gene cluster encodes proteins including XssABCDE (*Xanthomonas* siderophore synthesis) and XsuA (*Xanthomonas* siderophore utilization), which are homologous to PvsABCDE (*Vibrioferrin* biosynthesis) and PsuA (*Vibrioferrin* receptor) (Pandey and Sonti, 2010; Pandey et al., 2017). The *xss* gene loci involved in biosynthesis, uptake, and export of xanthoferrin is found in both *X. hawaiiensis* sp. nov. strains A6251^T^ and A2111. In addition, the *xanC* gene, which encodes an acyl carrier protein and is essential for yellow xanthomonadin pigment biosystem (Cao et al., 2018), is harbored in the genomes of A6251^T^ and A2111.

The increasing number of studies on non-pathogenic xanthomonads isolated from rice, banana, citrus, walnut, and so on suggests that they have the potential for biocontrol and bioprotection against the causal agent of their host plants (Fernandes et al., 2021; Bansal et al., 2021a,b; Rana et al., 2022). For example, *X. sontii* strain R1 (formerly misclassified as *X. sacchari*) isolated from rice seed was reported to have an antagonistic ability against *Burkholderia glumae*, which caused rice panicle blight disease (Xie et al., 2003; Ham et al., 2011; Fang et al., 2015). In addition, *Xanthomonas* sp. from ryegrass, which was phylogenetically closely related to *X. translucens*, showed bioprotection activities against broad tested fungal pathogens (Li et al., 2020). In the previous studies (Leite et al., 1994; Lee et al., 2020), the gene cluster involved in type III secretion system (T3SS) formation was amplified to identify the non-pathogenicity and pathogenicity strains of *X. campestris*. While deciphering the genomes of *X. hawaiiensis* sp. nov. strains, the T3SS gene cluster was missing in both genomes of A6251^T^ and A2111 (Chuang, 2023). The absence of T3SS was also observed in non-pathogenic strains of *X. campestris* (Lee et al., 2020), *X. sacchari* NCPPB 4393 and R1 strains (Studholme et al., 2011; Fang et al., 2015), and considerably commensal *X. arboricola* CFBP 6771 (Cesbron et al., 2015; Merda et al., 2017). Furthermore, based on 16S rRNA and nine housekeeping genes, the ML trees (Figures 2, 3) revealed that *X. hawaiiensis* sp. nov. strains A6251^T^ and A2111 were placed in Clade I along with *X. sacchari* and *X. traslucens*, which have potential biocontrol and bioprotective agents, as previously described. Hence, the genomic constituents of A6251^T^ and A2111 strains not only suggest that *X. hawaiiensis* sp. nov. strains isolated from Araceae should be commensal but also provide insight into the potential biocontrol capabilities of *X. hawaiiensis* sp. nov.

Recent research has begun to unravel the potential for biotechnological applications and biological control of stenotrophomonads. In agriculture, for example, *Stenotrophomonas* strains are known for promoting plant growth, protecting plants against biotic and abiotic stresses, and serving as biocontrol agents for plant diseases (Zhang and Yuen, 1999; Wolf, 2002; Messiha et al., 2007; Alavi et al., 2013; Berg and Martinez, 2015). As bioremediators and phytoremediators, *Stenotrophomonas* strains are capable of metabolizing and degrading a broad range of organic compounds, such as benzene and toluene, and tolerating antibiotics and heavy metals, such as mercury and silver (Binks et al., 1995; Alonso et al., 2000; Lee et al., 2002; Pages et al., 2008). Although *S. aracearum* sp. nov. A5588^T^ strain and *S. oahuensis* sp. nov. A5586^T^ strain showed no subsystem features of iron acquisition and metabolism, the higher number of RNA metabolism in A5586^T^ strain and protein metabolism in A5588^T^ strain (Figure 1B) might shed light on some unique metabolic activities in these new *Stenotrophomonas* species. Interestingly, the high number of unique genes and hypothetical protein encoding genes unraveled from detailed genomic contents of the novel species, especially in *S. oahuensis* sp. nov., imply that novel or useful enzymatic properties and metabolic capabilities of *Xanthomonas* and *Stenotrophomonas* spp. from different environmental sources are worth exploring for biocontrol and bioprotection purposes. Preliminary data from pathogenicity tests on anthurium indicated that strains A5588^T^ and A5586^T^ from anthurium are non-pathogenic stenotrophomonads due to lack of symptom development on their original host. In this study, we proposes three new species, namely, *X. hawaiiensis* sp. nov., *S. aracearum* sp. nov., and *S. oahuensis* sp. nov., isolated from Araceae and provides high quality whole genome sequences for further studies relative to their pathogenicity on Araceae host plants and other possible bioactivities.

## Data availability statement

The datasets presented in this study can be found in online repositories. The names of the repository/repositories and accession number(s) can be found at: https://www.ncbi.nlm.nih.gov/genbank/, CP115541-CP115542; https://www.ncbi.nlm.nih.gov/genbank/, CP115543; https://www.ncbi.nlm.nih.gov/genbank/, CP115873.

## Author contributions

S-CC: Data curation, Formal analysis, Investigation, Methodology, Software, Validation, Visualization, Writing – original draft, Writing – review & editing. SD: Methodology, Project administration, Supervision, Writing – review & editing. AA: Funding acquisition, Methodology, Resources, Writing – review & editing. MA: Conceptualization, Funding acquisition, Investigation, Project administration, Resources, Supervision, Validation, Visualization, Writing – review & editing.

## Footnotes

^1^ https://pacificbacterialcollection.com/

^2^ https://www.bv-brc.org/

^3^ http://www.geneious.com

^4^ https://itol.embl.de

^5^ https://tygs.dsmz.de/
